# Genome-wide association mapping and genomic prediction for late blight and potato cyst nematode resistance in potato (*Solanum tuberosum* L.)

**DOI:** 10.3389/fpls.2023.1211472

**Published:** 2023-10-04

**Authors:** Salej Sood, Vinay Bhardwaj, Aarti Bairwa, Dalamu, Sanjeev Sharma, Ashwani K. Sharma, Ashwani Kumar, Mehi Lal, Vinod Kumar

**Affiliations:** ^1^ Indian Council of Agricultural Research (ICAR)-Central Potato Research Institute, Shimla, HP, India; ^2^ ICAR-Central Potato Research Institute, Regional Station, Modipuram, UP, India

**Keywords:** GBS, snps, GEBV, genomic prediction, association mapping, late blight, PCN

## Abstract

Potatoes are an important source of food for millions of people worldwide. Biotic stresses, notably late blight and potato cyst nematodes (PCN) pose a major threat to potato production worldwide, and knowledge of genes controlling these traits is limited. A genome-wide association mapping study was conducted to identify the genomic regulators controlling these biotic stresses, and the genomic prediction accuracy was worked out using the GBLUP model of genomic selection (GS) in a panel of 222 diverse potato accessions. The phenotype data on resistance to late blight and two PCN species (*Globodera pallida* and *G*. *rostochiensis*) were recorded for three and two consecutive years, respectively. The potato panel was genotyped using genotyping by sequencing (GBS), and 1,20,622 SNP markers were identified. A total of 7 SNP associations for late blight resistance, 9 and 11 for *G. pallida* and *G*. *rostochiensis*, respectively, were detected by additive and simplex dominance models of GWAS. The associated SNPs were distributed across the chromosomes, but most of the associations were found on chromosomes 5, 10 and 11, which have been earlier reported as the hotspots of disease-resistance genes. The GS prediction accuracy estimates were low to moderate for resistance to *G*. *pallida* (0.04-0.14) and *G*. *rostochiensis* (0.14-0.21), while late blight resistance showed a high prediction accuracy of 0.42-0.51. This study provides information on the complex genetic nature of these biotic stress traits in potatoes and putative SNP markers for resistance breeding.

## Introduction

Potato (*Solanum tuberosum* L.) is a major commercial crop and is grown in over 100 countries worldwide. It is 3^rd^ in importance after rice and wheat (Sood et al., 2020a). Potatoes are a significant source of income for many farmers and contribute significantly to the economies of many countries. The global production of potatoes amounted to 370.43 million tonnes in 2019 from an area of 17.34 million hectares (FAOSTAT, 2021). In India, potatoes are believed to be introduced by Portuguese traders or by British missionaries (Pushkar, 1976). The first introduced cultivars, largely from European countries, were adapted to long-day and failed to express their yield potential under India’s sub-tropical short-day conditions. Based on locally adapted potato breeding programmes and the utilization of exotic South American landraces, the first suitable varieties were introduced to the market from the 1970s onward. Since then, the crop demand is continuously increasing, and India occupies 2nd place after China in potato production. Consistent efforts to improve crop productivity and quality have been hampered by crop genetics and various biotic and abiotic stresses. The potato has a low propagation coefficient and a long breeding cycle of about 12-13 years to develop new varieties (Jansky and Spooner, 2018; Sood et al., 2020a). Genetic gains in potato breeding have been low compared to major cereal crops in the last century (Sood et al., 2020b; Sood et al., 2022a; Sood et al., 2022b). Low genetic gains in the crop have been attributed to tetrasomic inheritance, heterozygous progenitors, clonal propagation through tubers and inability to recover the recipient genotype background after introgression of traits (Phumichai et al., 2022). Besides tuber yield, resistance to biotic stresses are the key traits in potato breeding programs, which farmers attach the highest importance to get good production (Kolech et al., 2017). Late blight caused by the oomycetes pathogen *Phytophthora infestans* (Mont.) de Bary is a major threat among biotic stresses due to pathogen virulence and adaptability (Chen et al., 2018; Ivanov et al., 2021). It is the most devastating disease worldwide, causing € 12 billion crop losses annually (Haverkort et al., 2009). In India, potatoes are grown from hills to plains in different agroecologies and seasons. Late blight is a major production constraint, particularly in hills, plateaus and the Eastern region of India, now started appearing above the threshold level in the Northern and Central Plains (Lal et al., 2018). Similarly, PCN caused by *Globodera* spp. is a major threat in temperate potato-growing areas (Chandel et al., 2020; Mburu et al., 2020). Moreover, PCN is a quarantine pest and restricts the movement of tubers from one place to the other. Understanding the genetics and identification of resistance genes is required to counter these biotic stresses in potatoes.

Both genome-wide association studies (GWAS) and genomic selection (GS) are based on linkage disequilibrium and are powerful genomic tools to target major quantitative trait loci (QTLs) and identify the best genetics lines, respectively, for target traits. Both GWAS and GS require high-density molecular markers, especially single nucleotide polymorphism (SNPs) spread across the genome. Although 8K, 12K and 20K SNP arrays have been available in potatoes (Hamilton et al., 2011; Vos et al., 2015), they were developed using specific germplasm. These arrays are, therefore, not suitable for genotyping Indian germplasm, which contains introductions from various countries and introgression lines developed through recombination breeding (Sood et al., 2021). *De-novo* genotyping using genotyping by sequencing (GBS) is the best alternative used previously in potatoes (Uitdewilligen et al., 2013; Sverrisdóttir et al., 2017). GWAS has been successfully used in different crops, including potatoes for late blight (Lindqvist-Kreuze et al., 2021; Wang et al., 2021), fry colour (Byrne et al., 2020) and various tuber traits (Sharma et al., 2018). GWAS captures a portion of genetic variance in the form of QTLs for complex quantitative traits, where QTLs with major effects are most likely identified, validated and can be used in marker-assisted selection. Although many important loci governing key traits have been identified using GWAS, GS has the advantage of capturing total genetic variance for the trait of interest, *i.e.*, genomic estimated breeding value (GEBV) and is a promising approach for selecting future individuals to improve the genetic gains in crop breeding (Sood et al., 2020c). GS has shown its promise in animal breeding and is introduced and tested in crop breeding programs (Daetwyler et al., 2014; Slater et al., 2016). Good prediction accuracy for key traits is essential for implementing GS in crop breeding. The results from different studies show low to high prediction accuracies for different traits, affected by various genetic factors such as trait heritability, relatedness, training population size and marker density. Stich and Van Inghelandt (2018) and Enciso-Rodriguez et al. (2018) observed moderate (0.4) to high (0.8) cross-prediction accuracies for various traits, including tuber yield, while Sood et al. (2020c) low (0.2) prediction accuracy for plant maturity. Besides genetic factors, the statistical models used for GS also affect the prediction accuracy. The choice of models is an important factor in implementing GS, and several parametric and non-parametric genomic prediction models are available. One of the most common and widely used parametric genomic selection models is the best linear unbiased prediction (BLUP). It is a mixed model–based whole-genome regression approach used to estimate the marker effects, which has been successfully applied to predict complex traits (Slater et al., 2016). Potato being auto-tetraploid and heterozygous makes it different from other pure-lines or inbred-based crops for implementing genomic selection. Few studies have been conducted to test the prediction accuracy of different traits in potato breeding (Sverrisdóttir et al., 2017; Byrne et al., 2020; Lindqvist-Kreuze et al., 2021) but still require more studies for better predictions and implement GS in potato breeding programs.

This study was conducted to generate genotype and phenotype data on late blight and PCN in the diverse potato panel for use in GWAS and Genomic Prediction (GP). The GWAS analysis was performed to identify novel QTL loci and explore the potential utility of GS in potato breeding for both biotic stresses.

## Materials and methods

### Plant materials and field trials

The plant material comprised 367 potato accessions, including Indian and exotic varieties and advanced breeding lines (**Table S1A**). The accessions were provided as *in-vitro* plants from the national active potato gene bank at ICAR-Central Potato Research Institute (CPRI), Shimla. The *in-vitro* plants were raised in the soil in a net-house from October-March under field conditions at CPRI, RS, Modipuram for tuber formation and multiplication during 2017-2018. The multiplied tubers were used for field evaluation at Kufri, Shimla, India. For the phenotype data, the number of accessions varied yearly for late blight and PCN resistance evaluation. Similarly, 288 accessions were used initially for genotyping and 222 could finally be considered based on the data quality (**Table S1B**). These 222 accessions were used for genetic diversity, GWAS and genomic prediction analysis.

For late blight evaluation, 10 tubers of each accession were planted at a spacing of 60×20cm in a single row of 2m during the summer (June-September) at Kufri, Shimla, Himachal Pradesh, India. Kufri is situated in the high hills of north-western Himalaya between 32°N, 77°E at an altitude of 2501m amsl. Kufri receives an average rainfall of 1520 mm annually with a temperature range of 9.1 (min) to 27.1°C (max) during potato crop season and is a natural hotspot for field screening of potato accessions to late blight resistance. The population of *Phytophthora infestans* in the experimental area is A2 mating type possessing 9-11 virulence genes (Sharma et al., 2016; Sharma et al., 2017). The field trials were conducted for three consecutive years from 2018 to 2020. The land preparation and fertiliser application were given as per the recommendations of the crop in the region. Pre-emergence herbicide application was given, followed by one manual weeding within a month after planting. The crop was specifically raised for recording the late blight incidence and most of the lines did not observe tuber formation due to high late blight incidence. Indian potato varieties, *viz*., ‘Kufri Jyoti’, ‘Kufri Himalini’ and ‘Kufri Girdhari’, were used as susceptible, moderately resistant and highly resistant genotypes, respectively. Late blight observations were recorded at weekly intervals after the first appearance of late blight on the susceptible check variety, Kufri Jyoti and continued till the susceptible control variety observed 100% disease incidence. Disease severity was evaluated as the percentage of foliage area of plants infected in the plot. Data were collected on each accession, and disease severity was recorded at weekly intervals. The area under the disease progress curve (AUDPC) was calculated as per the standard formula (Forbes et al., 2014; Sood et al., 2020b). The year-wise AUDPC values were used for GWAS and GP analyses.

For PCN resistance screening, 3 well-sprouted tubers of each accession were raised in pots using PCN infested soils during the summer (June-September) under controlled conditions in a glass house at Kufri, Shimla, Himachal Pradesh, India. Indian potato varieties *viz*., Kufri Jyoti and Kufri Himalini were used as susceptible controls. Phenotypic screening was done using the root-ball technique described by Sudha et al. (2016). The tubers were planted in pots (10 cm diameter) containing about 500g soil in glass house. The soil used for planting contained a mixed population of both PCN species, *i.e*., *Globodera pallida* and *G*. *rostochiensis* (200-250 cysts per 100 g soil), which provides 8000-10000 eggs and juveniles per test tuber. The root ball was examined for the presence of PCN females from the 55^th^ day after planting until the 65^th^ day. The two species were distinguished by the colour of developing females (White – *G*. *pallida*; Yellow- *G*. *rostochiensis*). Based on the number of females developed per root ball, the accessions were categorised into 0= Immune (Grade 0), 1 to 5 = Highly resistant (Grade 1), 6 to 20= Moderately resistant (Grade 2), 21 to 50 = Susceptible (Grade 3), and >50 = Highly susceptible (Grade 4). The planting was staggered (one week gap), accommodating 75 accessions at a time for proper maintenance and observations in the glass house. The evaluation was carried out for two years, *i.e*., 2020 and 2021 and the grades for each species individually were used for GWAS and GS analysis.

### GBS Library preparation and sequencing

Leaf samples were collected from field-grown plants of each accession and stored at -80°C for sequential DNA extraction in batches of 24 samples daily. The Doyle & Doyle (1987) CTAB technique was used to extract genomic DNA. The quality of the isolated DNA was checked using NanoDrop^®^ 2000 spectrophotometer as per the manufacturer’s instructions. Sample DNA with an OD260/OD280 ratio of 1.8 to 2.0 and a total quantity of more than 1.5ug was required for library construction. A 1% agarose gel was also used to evaluate DNA quality. Based on the outcomes of the *in-silico* evaluation, the 0.3~0.6 μg genomic DNA of each sample was digested with MseI and EcoRI double enzymes. The resulting fragments were ligated with P1 and P2 barcoded adapters with complementary sticky ends to the digested DNA and the Illumina P5 or P7 universal sequence. All the samples were pooled and size-selected for the necessary fragments to complete the library creation after multiple rounds of PCR amplification. Following cluster preparation, high-throughput DNA sequencing was performed on the Illumina HiSeq 2500 platform with a read length of 144 bp at each end (Elshire et al., 2011). **Figure S1** depicts the experimental procedure for DNA library preparation.

### Sequence alignment, SNP discovery and genotype calling

Initial fastq files were processed for read quality (Q>20) using a custom Perl script for trimming low-quality bases. The trimmed sequence data were aligned to the reference potato genome (Pham et al., 2020) (http://solanaceae.plantbiology.msu.edu/dm_v6_1_download.shtml) using Bowtie2 to obtain the SAM file. The SAM file was further converted into a BAM file for faster manipulation. The GATK (Genome Analysis Toolkit) was used for variant calling using the criterion described earlier (Caruana et al., 2019). The five genotype classes were assigned (AAAA, AAAB, AABB, ABBB, or BBBB) using the HaplotypeCaller function.

### LD and LD decay

In total, >1,000,000 SNPs were identified initially, which were reduced to 120,622 using missing data filters. The number was still too high for linkage disequilibrium (LD) decay and genomic prediction; therefore, the number was further reduced to 2,000 per chromosome using Unix and awk commands. We used a complete set of markers, *i.e*., 120,622 SNPs, for all analysis except LD decay, while a reduced set, *i.e*., 24,000 markers were used for LD decay, GWAS and GP only.

### Genetic diversity

The genetic diversity measures for each SNP, *i.e*., allele count, gene diversity or heterozygosity of the population (He), number of effective alleles in the population (Ne) and the polymorphic information content (PIC) were calculated using the vcfR tool (Knaus and Grünwald, 2017). A neighbor-joining dendrogram for all accessions was generated using TASSEL Version 5.0 Standalone (Bradbury et al., 2007).

### Genome-wide association study

GWAS was conducted using late blight AUDPC values recorded for three years, *i.e*., 2018-2020 and PCN resistance score on *G*. *rostochiensis* and *G*. *pallida* for two years, *i.e*., 2020 and 2021. The number of accessions evaluated varied yearly; therefore, each year’s analysis was carried out independently. R package GWASpoly, designed for GWAS with biallelic SNPs in autopolyploids using the Q (or P) + K method, was used for marker-trait analysis. Additive and simplex dominance marker effect models were considered to identify significantly associated SNPs with late blight and PCN. The default method, “M.eff” was used for a significant threshold, which is a Bonferroni-type correction using an effective number of markers that accounts for LD between markers (Moskvina and Schmidt, 2008). LD decay plot, QQ plot and Manhattan plot for each trait were also generated using the GWASpoly package (Rosyara et al., 2016).

### Identification of candidate genes

The candidate genes were identified within the associated genomic region in the potato genome using the significant marker-trait association results and the PGSC potato genome sequence portal (Pham et al., 2020; http://spuddb.uga.edu/dm_v6_1_download.shtml, accessed Oct 15, 2022). The genes that were in the ±50 bp window around the most significant SNPs were identified to ascertain the function.

### Genomic prediction

The genomic selection and prediction was evaluated using the GBLUP model (Meuwissen et al., 2001). Single-trait linear mixed models were used as follows:


y=1nμ+Zu+e

where, y is a vector of phenotypic records for the particular trait, μ is the overall mean, 1n is a vector of ones, Z is a matrix allocating records to breeding values, u is a vector of breeding values, and e is a vector of random error terms distributed as N(0, Iσ^2^_e_), and σ^2^_e_ is the error variance. The R package ‘Sommer’ was used to fit all genomic predictions (Covarrubias-Pazaran, 2016). The genomic relationship matrix was generated using the ‘AGHmatrix’ package in R (Amadeu et al., 2016; Slater et al., 2016). The genomic selection prediction accuracy was evaluated using fivefold cross-validation. For the genomic prediction of each group, the remaining four groups were used as a reference population. There were 50 repetitions of the random sample training and validation sets. Prediction accuracy was calculated as the Pearson correlation between the predicted genomic estimated breeding values (GEBVs) and the observed phenotypic values.

## Results

### Phenotypic data analysis

**Figure 1:**
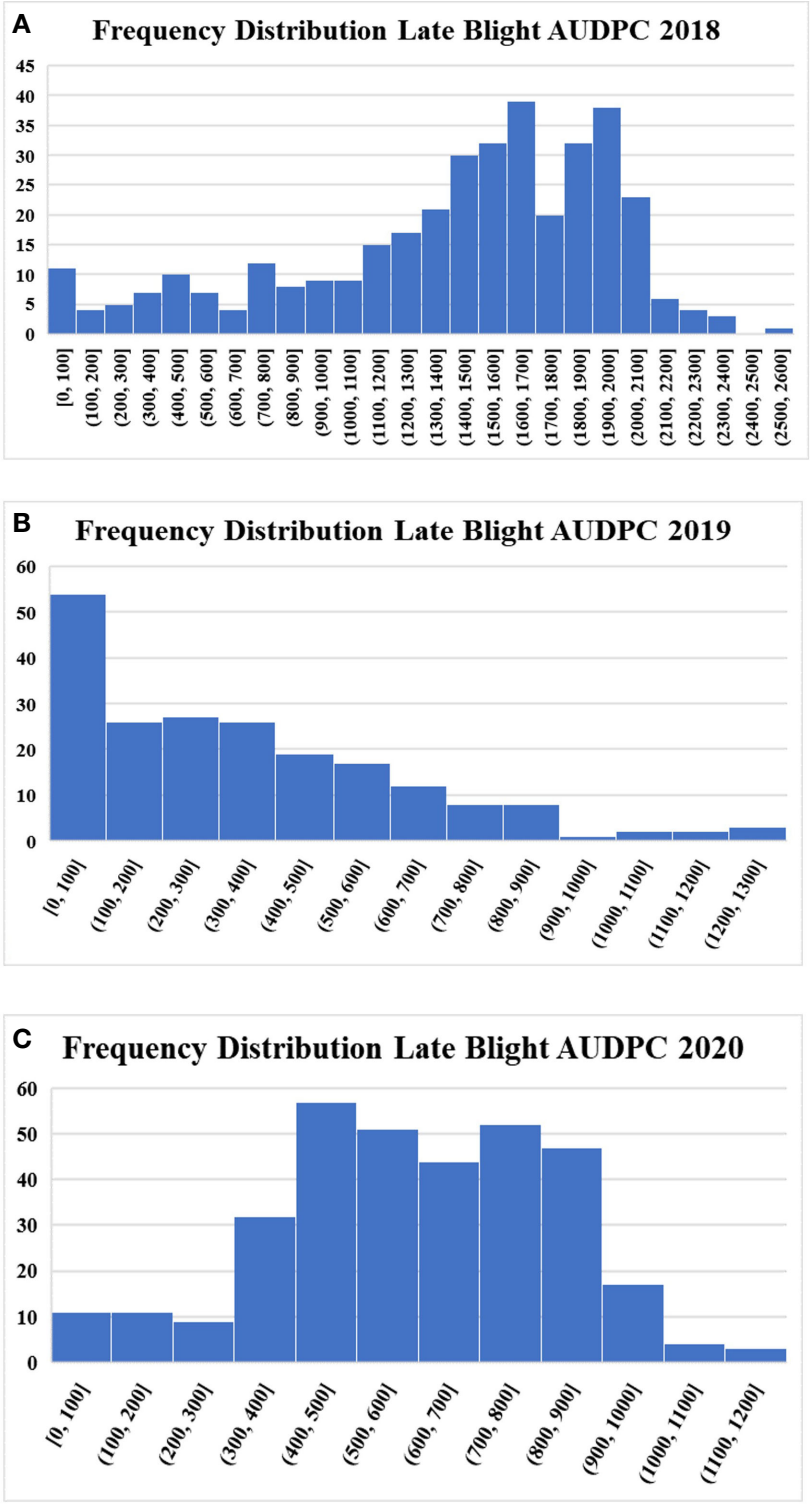
Frequency distribution of potato accessions for late blight resistance **(A)** Late blight AUDPC 2018 **(B)** Late blight AUDPC 2019 **(C)** Late blight AUDPC 2020.

In total, 205, 338, and 367 accessions were evaluated for late blight resistance in 2018, 2019, and 2020, respectively. Late blight AUDPC values varied from 0 to 2520, 0 to 1265 and 0 to 1192 in three different years. The susceptible control variety Kufri Jyoti recorded an AUDPC value of 2060 (2018), 707.5 (2019) and 810 (2020), while the resistant variety Kufri Girdhari did not observe any disease incidence and recorded zero AUDPC value across the years. The moderately resistant variety Kufri Himalini observed an AUDPC of 840 (2018), 572.5 (2019) and 315 (2020) during three different years. Overall, we observed a wide range of variation from highly resistant to highly susceptible accessions for late blight in the population (**Figure 1**). The frequency distribution for the late blight AUDPC was near normal for 2018 and 2020, but 2019 observed a left-skewed distribution. The relationship between AUDPC values of accessions across the years is presented in **Figure S2**. The AUDPC values showed strong linear relationship in different years as depicted by the correlation values of 0.63 (AUDPC 2018 & AUDPC 2019), 0.68 (AUDPC 2018 & AUDPC 2020) and 0.66 (AUDPC 2019 & AUDPC 2020).

**Figure 2:**
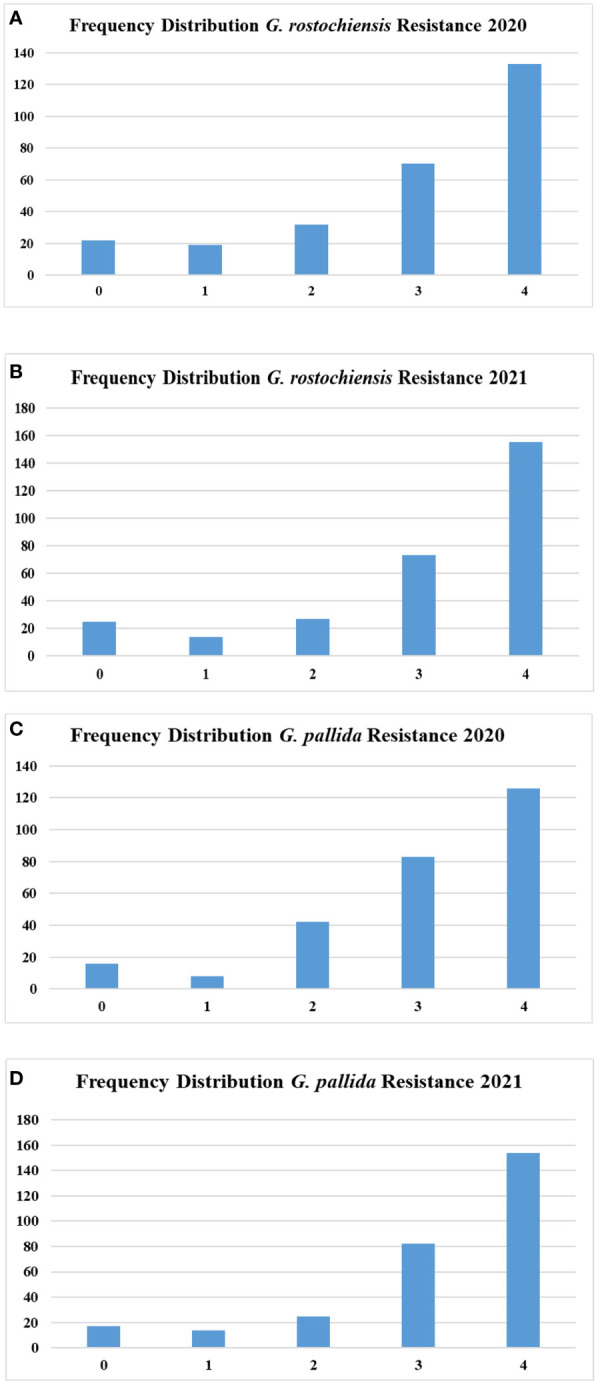
Frequency distribution of potato accessions for potato cyst nematode resistance **(A)** Resistance to *G*. *rostochiensis* in 2020 **(B)** Resistance to *G*. *rostochiensis* in 2021 **(C)** Resistance to *G*. *palida* in 2020 **(D)** Resistance to *G*. *palida* in 2021.

For PCN evaluation, the scoring was performed for both the species, *i.e*., *G. rostochiensis* and *G*. *pallida*, in three replications and a mean value was used for analysis. The number of accessions under evaluation was 278 and 294 in 2020 and 2021, respectively. We observed highly resistant to highly susceptible accessions in the population for both species in both evaluation years. The susceptible controls *viz*., Kufri Jyoti and Kufri Himalini observed highly susceptible reaction (grade - 4) to both the species of the PCN during both the evaluation years. The number of accessions under each category are shown in **Figure 2**. The accessions under the highly resistant and resistant categories were less in number than susceptible and highly susceptible accessions. The scoring of accessions across the years showed good relationship as depicted in **Figure S3**. The correlation between PCN scores of two different years was 0.76 and 0.79 for *G*. *rostochiensis* and *G*. *pallida*, respectively.

### Genotyping and SNP markers

An average of 3.3 million reads per sample were generated for 222 tetraploid potato accessions, with a range of 1.5 - 5.2 million reads. Over 94% of the filtered high-quality reads were aligned to the potato reference genome (Pham et al., 2020). Initially, a set of 1,024,680 SNPs were detected, which were reduced to 120,622 high-confidence SNPs following missing data filters (site coverage ≥90%) and minor allele frequency (MAF ≥ 0.05). The SNPs were evenly distributed with an average density of 1.5 SNPs/10kb region and were proportional to the chromosome size for all the 12 chromosomes (**Table 1**). The minimum number of SNPs were present on the smallest chromosome, *i.e*., Chr. 11 (6,538), while the highest was on the largest chromosome, *i.e*., Chr. 1 (13,610) (**Table 1**; **Figure S4**).

**Table 1:** Distribution of filtered SNPs and SNP density across chromosomes.

Chromosome	Length (in bp)	Number of SNPs detected	SNP Density/10Kb
Chr01	8,86,63,952	13610	1.5
Chr02	4,86,14,681	7524	1.5
Chr03	6,22,90,286	11952	2.0
Chr04	7,22,08,621	11988	1.6
Chr05	5,20,70,158	7594	1.5
Chr06	5,95,32,096	11945	2.0
Chr07	5,67,60,843	8038	1.4
Chr08	5,69,38,457	8724	1.5
Chr09	6,15,40,751	12752	2.1
Chr10	5,97,56,223	12611	2.1
Chr11	4,54,75,667	6538	1.4
Chr12	6,11,65,649	7346	1.2

**Figure 3:**
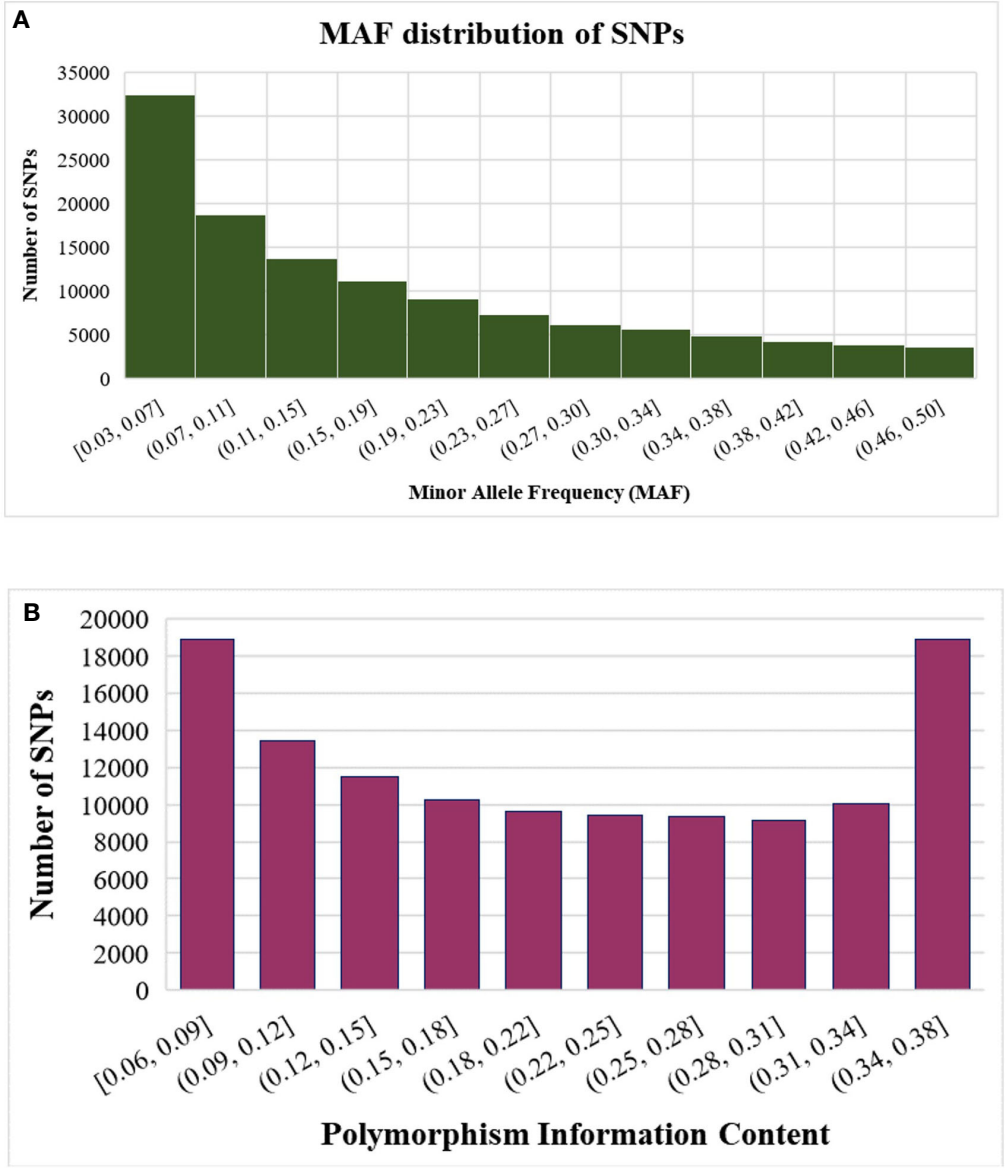
Statistics of SNPs identified using GBS **(A)** Distribution of SNPs based on minor allele frequency (MAF) **(B)** Distribution of SNPs based on polymorphism information content (PIC).

Besides trait phenotype diversity, the germplasm accessions also displayed wide genetic diversity, which reflect their diverse geographic origin, market use type, and target areas. The MAF distribution of SNPs showed enrichment for SNPs with higher MAF (>0.07) across all accessions desirable for performing GWAS (**Figure 3A**).

Genetic diversity in the germplasm accessions was assessed using estimates of marker PIC values. The PIC values of the SNPs ranged from 0.06 to 0.38 with a mean value of 0.21 (**Figure 3B**). Most of the SNPs were highly polymorphic, with 48,294 SNPs (40%) showing PIC values ≥0.25 and none falling below 0.06 (**Figure 3B**).

**Figure 4:**
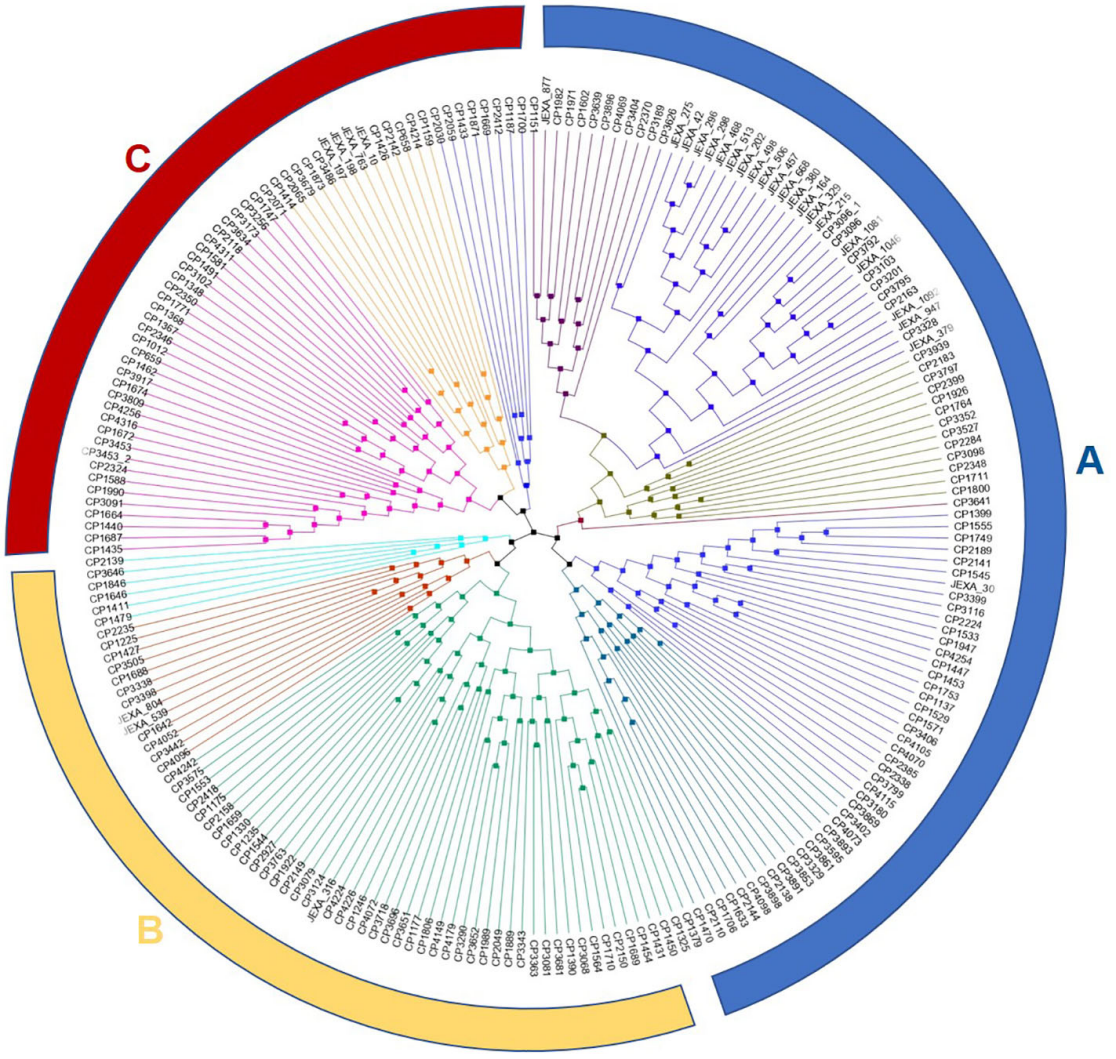
Neighbor-joining (NJ) phylogenetic tree displaying the genetic relationships among the 222 potato accessions in the panel based on 1,20,622 SNP markers. Branch lengths indicate genetic divergence i.e., the longer the branch, the more diverse the accession from other accessions.

The dendrogram generated through the neighbor-joining method grouped the accessions/varieties into three major clusters (A, B and C). Each major cluster was further subdivided into two clusters. The number of accessions/varieties in each cluster was 98, 67 and 57, respectively. Although there was no clear clustering pattern, most Indian varieties released for cultivation in the sub-tropical plains of India were grouped in cluster A with few exceptions. Similarly, the Andigena accessions were also grouped in a small sub-cluster in cluster A (**Figure 4**). The accessions from Australia, New Zealand and Afganistan were also grouped in cluster A. The cluster B and C had mixed accessions of Europe, North America and South America.

### LD and LD decay

**Figure 5:**
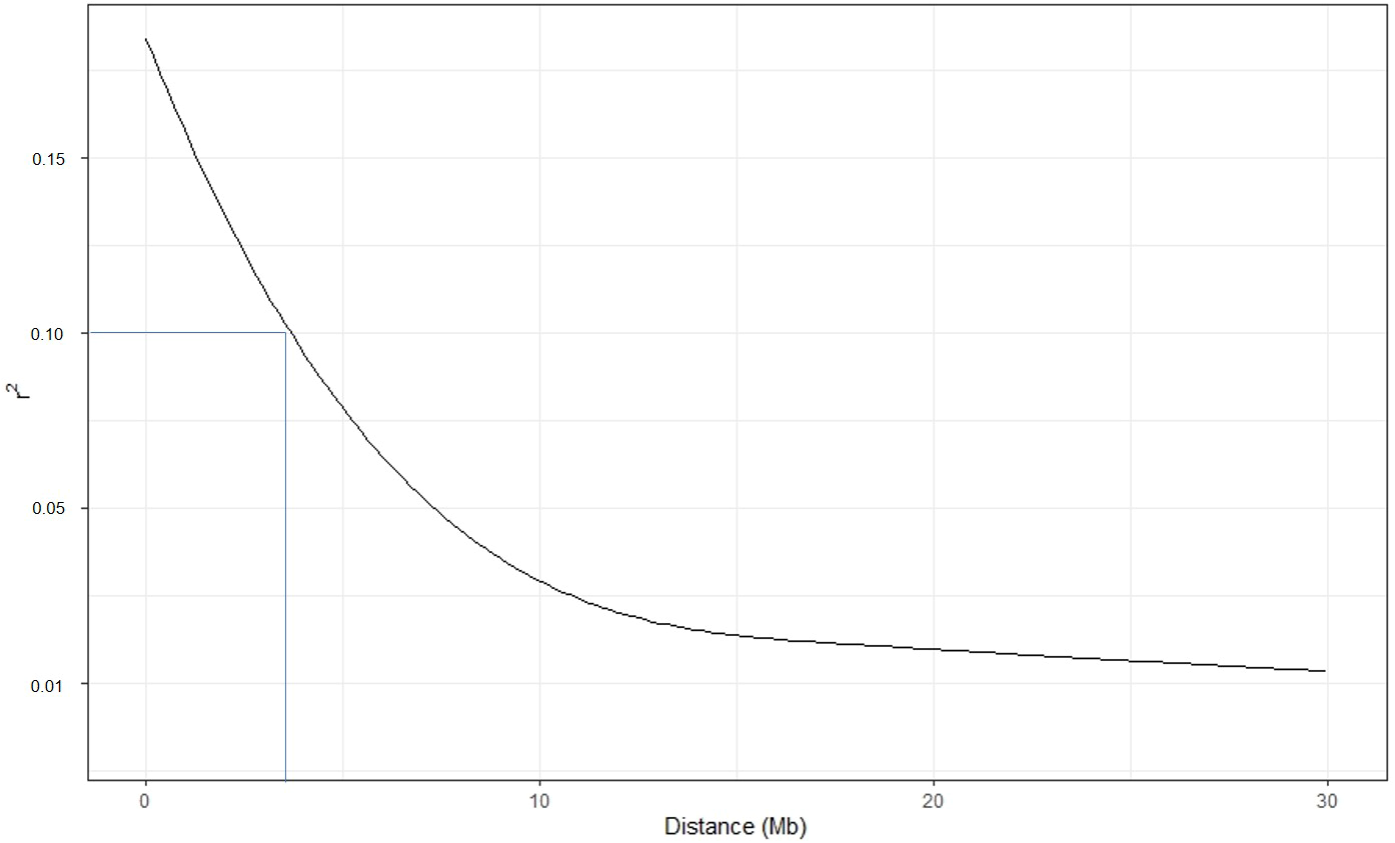
Linkage disequilibrium (LD) decay estimated in the potato accessions. The Pearson correlation coefficient was plotted against the physical map distance (Mb) between pairs of SNPs using a reduced marker data set (24000 SNPs across the genome).

To calculate the LD decay value, we reduced the number of SNPs per chromosome to 2,000 and the total number to 24,000. The pairwise r^2^ values were plotted with the physical distance of the markers. At r^2^ value 0.1, the LD decay was ~4Mb (**Figure 5**). The average LD was inconsistent across the chromosomes. High disequilibrium was observed on chromosome 10, followed by chromosomes 11 and 3 (**Figure S5**).

### GWAS analysis

**Figure 6:**
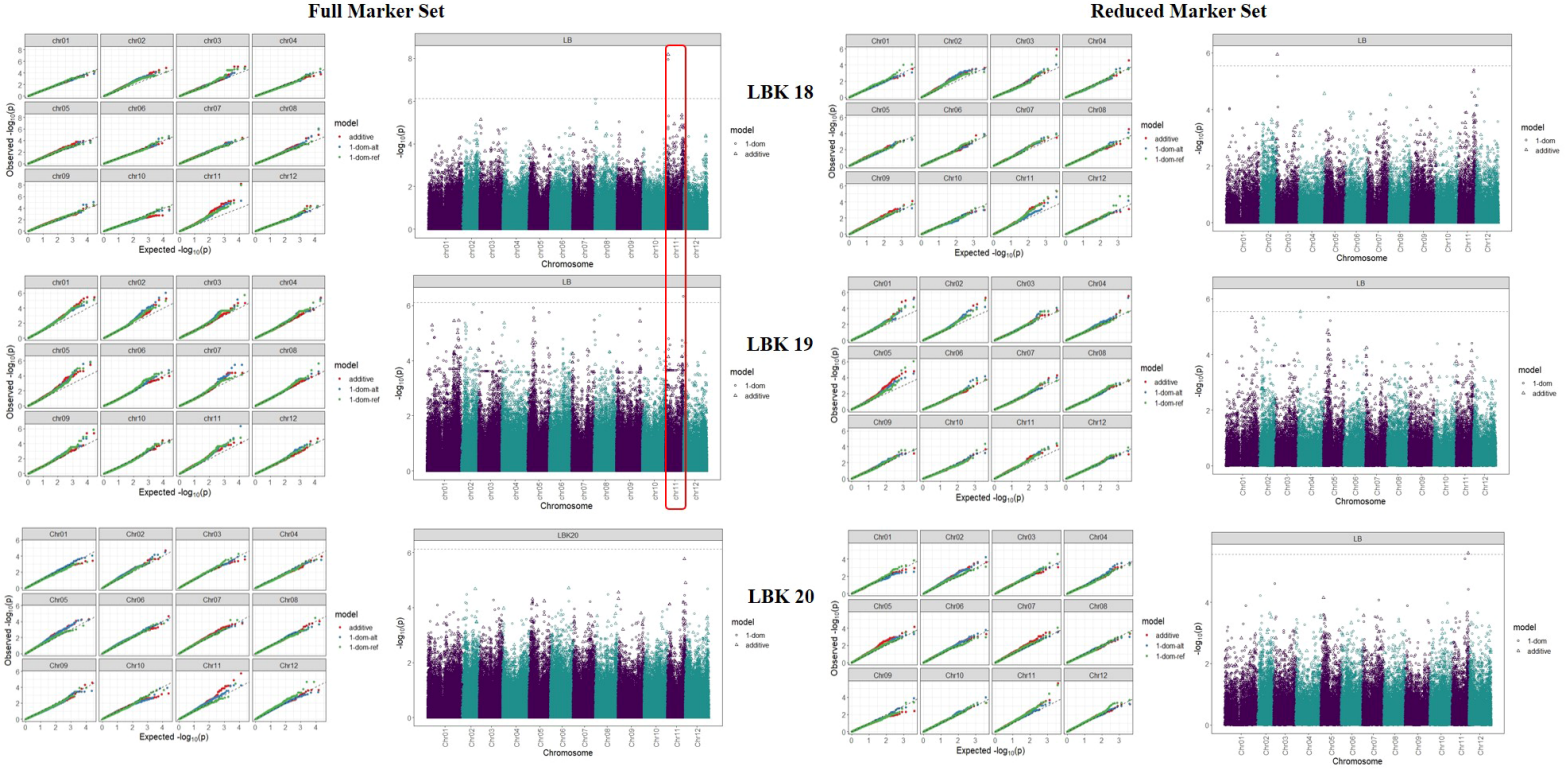
Q-Q plots comparing the inflation of p-values for both the traits using the additive and simplex dominance marker model in full vs reduced marker data sets. The black dotted line indicates p-values under the expected normal distribution.

We analysed the marker-trait associations (MTA’s) for auto-tetraploid potato genotypes using GWASpoly R package modelling additive and simplex dominance marker effects. For late blight resistance, 3 and 1 associations were found in 2018 and 2019 using the complete set of markers (**Table 2**; **Figure 6**). Out of these four markers, one was additive (chr11_5176739) with 15.6% phenotypic variance, while the other 3 were dominant alternate (chr08_1111466, chr11_5176739, chr11_45392300) with each QTL contributing to the phenotypic variance ranging from 12.3 - 17.1% (**Table 2**). The year 2020 data did not show any association of genotype with phenotype data for late blight resistance. However, reduced marker set data showed three additive QTLs, one each in 2018, 2019 and 2020, on chromosome 3, chromosome 4 and chromosome 11, respectively (**Table 2**). The phenotypic variance explained by these three additive QTLs varied from 10.5 - 12.7%. One dominant reference QTL was also found on chromosome 5 in 2019, which explained 9.9% phenotypic variance (**Table 2**). The results showed that chromosome number and position governing late blight resistance varied in the full vs reduced markers dataset.

**Table 2:** SNP markers significantly associated with the late blight and potato cyst nematode resistance phenotype in different years using complete and reduced marker data set.

Trait	Model	Marker	Chr	Position	Ref allele	Alt allele	Threshold	Score	Effect	R^2^	P value
Full marker set											
AUDPC18	additive	chr11_5176739	Chr11	5176739	T	G	6.12	8.19	-365.24	0.156	0
AUDPC18	1-dom-alt	chr08_1111466	Chr08	1111466	T	G	6.1	6.21	454.94	0.123	0
AUDPC18	1-dom-alt	chr11_5176739	Chr11	5176739	T	G	6.12	8.19	-365.24	0.156	0
AUDPC19	1-dom-alt	chr11_45392300	chr11	45392300	A	T	6.1	6.33	-741.63	0.171	0
GP20	additive	chr03_30051088	chr03	30051088	A	G	6.12	7.35	0.69	0.157	0
GP21	1-dom-alt	chr01_79870255	chr01	79870255	C	T	6.1	6.38	-1.72	0.078	0
GP21	1-dom-ref	chr05_39730271	chr05	39730271	C	T	6.1	6.37	-2.65	0.04	0.012
GP21	1-dom-ref	chr10_29357976	chr10	29357976	C	T	6.1	7.6	-1.95	0.058	0.003
GR20	1-dom-alt	chr01_48615182	chr01	48615182	C	T	6.11	6.69	-1.51	0.066	0
GR20	additive	chr03_27049362	chr03	27049362	A	G	6.12	7	0.91	0.115	0
GR20	1-dom-ref	chr05_52055419	chr05	52055419	A	G	6.11	6.21	1.30	0.045	0.003
GR20	additive	chr12_53396900	chr12	53396900	A	G	6.12	6.86	-0.80	0.048	0.003
GR20	additive	chr12_619482	Chr12	619482	A	G	5.55	6.41	-0.42	0.083	0
GR21	1-dom-alt	chr04_43994066	chr04	43994066	A	G	6.1	6.26	1.72	0.095	0
GR21	1-dom-alt	chr10_11705153	chr10	11705153	C	T	6.1	6.23	2.59	0.217	0
Reduced marker set											
AUDPC18	additive	chr03_2054715	Chr03	2054715	G	T	5.55	5.58	-298.04	0.105	0
AUDPC19	additive	chr04_5755401	Chr04	5755401	C	T	5.54	5.55	-125.50	0.127	0
AUDPC19	1-dom-ref	chr05_13326227	Chr05	13326227	A	G	5.53	6.06	-263.30	0.099	0
AUDPC20	additive	chr11_44083171	Chr11	44083171	A	G	5.55	5.6	50.96	0.107	0
GP20	additive	chr01_62889722	Chr01	62889722	C	T	5.55	5.83	-0.40	0.128	0
GP20	1-dom-ref	chr10_29996650	Chr10	29996650	A	G	5.54	5.73	-1.52	0.121	0
GP21	1-dom-alt	chr10_45333027	Chr10	45333027	A	G	5.53	7.44	2.84	0.024	0.053
GP21	1-dom-ref	chr10_29358030	Chr10	29358030	A	G	5.53	6.33	-1.88	0.065	0.001
GP21	1-dom-alt	chr11_43021827	Chr11	43021827	C	T	5.53	5.84	-1.27	0.065	0.001
GR20	1-dom-alt	chr01_14013398	Chr01	14013398	A	G	5.54	5.6	-1.42	0.109	0
GR20	additive	chr02_43988105	Chr02	43988105	A	T	5.55	5.69	0.69	0.115	0
GR20	1-dom-alt	chr05_47262811	Chr05	47262811	C	T	5.54	5.83	-1.66	0.065	0
GR20	additive	chr12_619482	Chr12	619482	A	G	5.55	6.41	-0.42	0.083	0
GR21	additive	chr10_29388315	Chr10	29388315	A	G	5.55	5.64	-0.66	0.088	0
GR21	1-dom-alt	chr03_23982469	Chr03	23982469	A	G	5.53	6.81	-2.07	0.129	0

Chr, Chromosome; Ref,Reference; Alt-Alternate; 1-dom-alt, dominant alternate; 1-dom-ref, dominant reference; AUDPC18, Late Blight 2018; AUDPC19, Late Blight 2019; AUDPC20, Late Blight 2020; GP20, Globodera pallida 2020; GP21, Globodera pallida 21; GR20, Globodera rostochiensis 20; GR21, Globodera rostochiensis 21.

**Figure 7:**
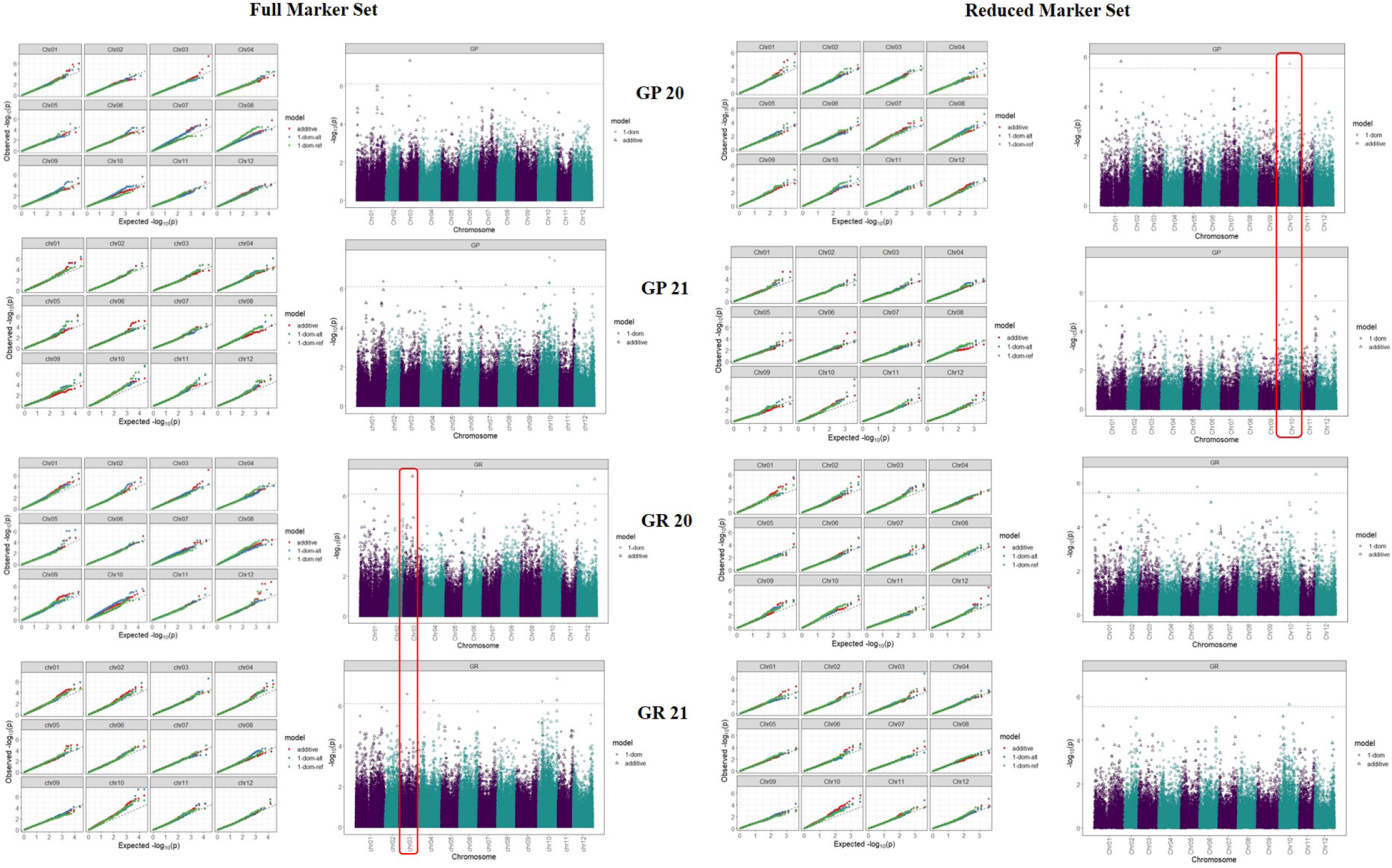
Manhattan plots for different traits in full vs reduced marker data sets. The significance threshold (black dashed line) is based on the genome-wide false positive rate (a = 0.05) for the Bonferroni correction method and the marker-trait associations (MTAs) crossing the set threshold are depicted in triangles and circles.

The PCN resistance markers were identified through separate GWAS analyses using phenotype data on both the PCN species, *i.e*., *G*. *pallida* and *G*. *rostochiensis*. The complete set of markers showed five MTAs for *G*. *rostochiensis* (3 additive, 1 dominant alternate and 1 dominant reference) and one for *G*. *pallida* (additive) in 2020. The *G*. *rostochiensis* markers, chr03_27049362 (additive), chr12_619482 (additive), chr12_53396900 (additive), chr01_48615182 (dominant alternate), and chr05_52055419 (dominant reference) showed a range of phenotypic variance (4.5 - 11.5%). The sole additive marker for *G*. *pallida*, chr03_30051088, explained 15.7% phenotypic variance in 2020. The year 2021 observed 2 MTAs for *G*. *rostochiensis* (dominant alternate) and 3 for *G*. *pallida* (1 dominant alternate and 2 dominant reference) in 2021 (**Table 2**; **Figure 7**). The *G*. *rostochiensis* markers, chr04_43994066 and chr10_11705153 explained a phenotypic variance of 9.5 and 21.7%, respectively, while *G*. *pallida* markers chr01_79870255, chr05_39730271 and chr10_29357976 showed a phenotypic variance range of 4.0-7.8%. The reduced marker set recorded 4 and 2 MTAs for *G*. *rostochiensis* in 2020 and 2021, respectively. The additive markers were present on chromosomes 2, 10 and 12 and explained a phenotypic variance of 8.2 to 11.5%, while dominant alternate markers were on chromosomes 1, 3 and 5 with phenotypic variance ranging from 6.5 to 12.9%. We found 2 and 3 MTAs for *G*. *pallida* in 2020 and 2021, respectively. The *G*. *pallida* MTAs were on chromosomes 1, 10 and 11, which explained 6.5 12.8% phenotypic variance (**Table 2**). For late blight resistance, we found major QTLs on chromosome 11, while PCN resistance QTLs were observed on chromosomes 3 and 10. The candidate genes corresponding to the QTLs for both traits are also presented in **Table 3**. The major genes for late blight resistance were functionally related to response regulators, proteins of unknown function, plant U-box and ENTH/VHS/GAT family protein. Similarly, the putative function of candidate genes for PCN resistance (*G. pallida and G. rostochiensis*) was found associated with intergenic region, hypothetical protein and response regulator.

**Table 3:** Candidate genes and their putative function of associated SNPs for late blight and PCN.

Trait	Marker	Candidate gene(s)	Putative role/function
AUDPC18	chr11_5176739	Soltu.DM.11G005070.1	“Protein of unknown function DUF455”
		Soltu.DM.11G005070.2	“Protein of unknown function DUF455”
		Soltu.DM.11G005080.1	“S-adenosyl-L-methionine-dependent methyltransferases superfamily protein”
		Soltu.DM.11G005080.2	“S-adenosyl-L-methionine-dependent methyltransferases superfamily protein”
	chr08_1111466	Soltu.DM.08G000610.1	“Polynucleotidyl transferase, ribonuclease H-like superfamily protein”
		Soltu.DM.08G000600.1	“RGPR-related”
	chr03_2054715	Soltu.DM.03G002130.1	“P-loop containing nucleoside triphosphate hydrolases superfamily protein”
AUDPC19	chr11_45392300	Soltu.DM.11G025400.1	“Response regulator”
		Soltu.DM.11G025400.2	“Response regulator”
		Soltu.DM.11G025400.3	“Response regulator”
		Soltu.DM.11G025410.1	“RNA-binding (RRM/RBD/RNP motifs) family protein”
		Soltu.DM.11G025390.1	“Hypothetical protein”
	chr04_5755401	Soltu.DM.04G005260.1	“Dentin sialophosphoprotein-related”
		Soltu.DM.04G005260.2	“Dentin sialophosphoprotein-related”
		Soltu.DM.04G005260.3	“Dentin sialophosphoprotein-related”
		Soltu.DM.04G005260.4	“Dentin sialophosphoprotein-related”
	chr05_13326227	Soltu.DM.05G011500.1	“Plant U-box”
		Soltu.DM.05G011490.1	“P-loop containing nucleoside triphosphate hydrolases superfamily protein”
AUDPC20	chr11_44083171	Soltu.DM.11G023910.1	“ENTH/VHS/GAT family protein”
GP20	chr03_30051088	intergenic region (MODIFIER)	“Intergenic region”
	chr01_62889722	Soltu.DM.01G023890.1	“Ribosomal protein L34e superfamily protein”
	chr10_29996650	intergenic region (MODIFIER)	“Intergenic region”
GP21	chr01_79870255	Soltu.DM.11G005070.1	“Protein of unknown function DUF455”
		Soltu.DM.01G041380.1	“Suppressor of auxin resistance1”
		Soltu.DM.01G041380.2	“Suppressor of auxin resistance1”
		Soltu.DM.01G041380.3	“Suppressor of auxin resistance1”
	chr05_39730271	intergenic region (MODIFIER)	“Intergenic region”
	chr10_29357976	intergenic region (MODIFIER)	“Intergenic region”
	chr10_45333027	Soltu.DM.10G016060.1	“Hypothetical protein”
	chr10_29358030	intergenic region (MODIFIER)	“Intergenic region”
	chr11_43021827	Soltu.DM.11G022980.1	“SIT4 phosphatase-associated family protein”
		Soltu.DM.11G022980.2	“SIT4 phosphatase-associated family protein”
		Soltu.DM.11G022980.3	“SIT4 phosphatase-associated family protein”
		Soltu.DM.11G022980.4	“SIT4 phosphatase-associated family protein”
		Soltu.DM.11G022970.1	“Auxin response factor”
		Soltu.DM.11G022970.2	“Auxin response factor”
		Soltu.DM.11G022970.3	“Auxin response factor”
		Soltu.DM.11G022970.4	“Auxin response factor”
GR20	chr01_48615182	Soltu.DM.01G018520.1	“Ubiquitin protein ligase”
	chr03_27049362	Soltu.DM.03G010020.1	“Response regulator”
	chr05_52055419	Soltu.DM.05G023650.1	“Cellulose synthase-like D1”
		Soltu.DM.05G023660.1	“Methionine–tRNA ligase, putative/methionyl-tRNA synthetase, putative/MetRS, putative
		Soltu.DM.05G023640.1	cellulose synthase-like D1”
	chr12_53396900	Soltu.DM.12G023490.1	“NB-ARC domain-containing disease resistance protein”
		Soltu.DM.12G023500.1	“NB-ARC domain-containing disease resistance protein”
		Soltu.DM.12G023500.2	“NB-ARC domain-containing disease resistance protein”
	chr12_619482	Soltu.DM.12G000550.1	“Phosphate transporter 4;6”
		Soltu.DM.12G000560.1	“Putative glycosyl hydrolase of unknown function (DUF1680)”
		Soltu.DM.12G000560.2	“Putative glycosyl hydrolase of unknown function (DUF1680)”
		Soltu.DM.12G000560.3	“Putative glycosyl hydrolase of unknown function (DUF1680)”
		Soltu.DM.12G000560.4	“Putative glycosyl hydrolase of unknown function (DUF1680)”
	chr01_14013398	intergenic region (MODIFIER)	“Intergenic region”
	chr02_43988105	Soltu.DM.02G032090.1	“Phosphoglycerate mutase-like family protein”
		Soltu.DM.02G032090.2	“Phosphoglycerate mutase-like family protein”
	chr05_47262811	Soltu.DM.05G019450.1	“Myb domain protein”
		Soltu.DM.05G019440.1	“Remorin family protein”
GR21	chr04_43994066	intergenic region (MODIFIER)	“Intergenic region”
	chr10_11705153	intergenic region (MODIFIER)	“Intergenic region”
	chr10_29388315	intergenic region (MODIFIER)	“Intergenic region”
	chr03_23982469	intergenic region (MODIFIER)	“Intergenic region”

AUDPC18, Late Blight 2018; AUDPC19, Late Blight 2019; AUDPC20, Late Blight 2020; GP20, Globodera pallida 2020; GP21, Globodera pallida 21; GR20, Globodera rostochiensis 20; GR21, Globodera rostochiensis 21.

### Genomic prediction

Genomic prediction accuracy for late blight and PCN resistance was evaluated using the GBLUP model in full vs reduced marker data sets (**Table 4**). The training population size was 107-152 for late blight resistance in three different years, while it varied from 131-134 for PCN resistance in two different years. The complete marker data set showed high heritability (0.55-0.84) for both traits across the years of evaluation. The genomic prediction accuracy estimates were low to negligible for *G*. *pallida* resistance (0.04 ± 0.011 to 0.10 ± 0.014), low to moderate for *G*. *rostochiensis* resistance (0.14 ± 0.011 to 0.21 ± 0.019) and high for late blight resistance (0.42 ± 0.017 to 0.51 ± 0.012) (**Table 4**).

**Table 4:** Late blight and PCN resistance prediction accuracy of full vs reduced marker data using the GBLUP model.

Trait	Training population size	Heritability (ns)		Cross Validation Prediction accuracy (rOPV : GEBV)			
				Range		Average ± S.E.*	
		FMS	RMS	FMS	RMS	FMS	RMS
GP20	134	0.55	0.38	-0.14-0.25	-0.23-0.31	0.10 ± 0.014	0.11 ± 0.016
GP21	131	0.60	0.46	-0.15-0.19	-0.15-0.33	0.04 ± 0.011	0.07 ± 0.016
GR20	134	0.82	0.63	-0.13-0.44	-0.13-0.50	0.21 ± 0.019	0.20 ± 0.017
GR21	131	0.70	0.65	-0.05-0.32	-0.10-0.39	0.14 ± 0.011	0.18 ± 0.018
AUDPC18	152	0.84	0.86	0.20-0.67	0.20-0.68	0.45 ± 0.015	0.46 ± 0.015
AUDPC19	107	0.80	0.80	0.34-0.68	0.33-0.69	0.51 ± 0.012	0.51 ± 0.012
AUDPC20	149	0.75	0.75	0.19-0.68	0.19-0.69	0.42 ± 0.017	0.43 ± 0.018

FMS, Full marker set; RMS, Reduced marker set; GEBV, Genomic estimated breeding value; OPV, Observed phenotype value; r, Pearson correlation; SE, Standard error; ns, Narrow sense; *, values are mean ± s.e. for 50 replicates.

The trait heritability based on the reduced marker data set was low for *G*. *pallida* (0.38-0.46) and high for *G*. *rostochiensis* (0.63-0.65) and late blight resistance (0.75-0.86). The genomic prediction accuracy also followed the heritability trend. *G*. *pallida* showed the lowest prediction accuracy (0.07 ± 0.016 to 0.11 ± 0.016), followed by *G*. *rostochiensis* (0.18 ± 0.018 to 0.20 ± 0.017), while the highest genomic prediction accuracy of 0.43 ± 0.018 to 0.51 ± 0.012 was observed for late blight using the GBLUP (**Table 4**).

The genomic prediction accuracy results with complete vs five times reduced marker data set showed almost similar results except for *G*. *pallida* and *G*. *rostochiensis* in 2021. In both cases, the prediction accuracy was perfect for late blight resistance across the years, while PCN resistance for the two species had low prediction accuracy.

## Discussion

Being a heterozygous and auto-tetraploid crop, the trait inheritance in potatoes is quite complex. The study identified several resistant elite donor lines for late blight and PCN, which could be used in introgression breeding to breed better varieties for Indian hills and plains. However, knowledge of the candidate genes/QTLs governing resistance could accelerate the breeding process and enhance the genetic gain in potato breeding. Therefore, we regenerated a diverse panel of 367 potato varieties and accessions from *in-vitro* collection of the ICAR-CPRI, Shimla, India and multiplied at CPRI Regional Station, Modipuram, India to investiagte the late blight and PCN. The phenotype data on late blight and PCN (both species) collected at the CPRI, Regional Station, Kufri, Shimla, India, showed wide variation among accessions for resistance level to both the biotic stresses. The frequency distribution histograms for late blight AUDPC showed that the number of resistant and highly susceptible accessions was low, whereas the maximum number of accessions were in the susceptible category except in 2019. In the case of the PCN resistance, both species showed very few numbers of resistant accessions, while the number of susceptible and highly susceptible accessions were very high. The results substantiate earlier studies on late blight and PCN resistance screening which state that several varieties resistant to late blight have been developed (Duan et al., 2021), but most popular cultivars do not possess resistance to both the species of PCN (Price et al., 2021). However, the existence of all categories from high resistance to high susceptibility, shows the suitability of the panel for genetic analysis, genome-wide association study and genomic prediction.

Further, the enrichment of common SNPs with high MAF and polymorphism of SNP markers (0.06-0.38; mean-0.21) revealed the suitability of selected SNPs for the genetic analysis of potato accessions. A higher average PIC value of 0.35 was reported earlier in cultivated European tetraploid potatoes genotyped with the SolCAP 8k SNP platform. The high average PIC value of SNPs in these populations could be attributed to the low total marker number and the genotyping platform used in these studies (Stich et al., 2013 and Sharma et al., 2018). However, the observed PIC values of the markers in the studied germplasm collection indicate high levels of polymorphism, further confirming the suitability of these SNP markers for genetic analysis of potato germplasm. Since the SolCAP SNPs have been derived from only six potato cultivars mainly representing North America, we used the GBS approach to avoid ascertainment bias and extend the application of derived SNPs across the global germplasm (Caruana et al., 2019).

As observed earlier, many SNPs across the population revealed high sequence diversity in potatoes (Hamilton et al., 2011; Uitdewilligen et al., 2013 and Caruana et al., 2019). An average density of 1.5 SNPs/10kb region across the genome showed high marker density, sufficient for GWAS and genomic selection in potatoes to capture the genetic variance for the trait of interest. Similar results on SNP density (one SNP every 17,469 bases) were observed earlier by Byrne et al. (2020). The phylogenetic analysis of the accessions using SNP data also showed wide diversity in the material under study.

The genome-wide linkage disequilibrium analysis using all 120,622 SNPs for tetraploid allele dosage is difficult to perform due to computational problems; therefore, we performed chromosome-wise LD analysis of a reduced marker set, *i.e*., 2,000 evenly distributed SNPs of each chromosome. LD decay determines the minimum marker number for GWAS and GS and the feasibility to tag QTLs for traits of interest. Crops with faster LD decay require very dense markers, whereas moderate to low LD decay crops need low marker density (Lindqvist-Kreuze et al., 2021). Moderate LD decay at a distance of 70Kb to 2Mb has been reported in the cultivated gene pool of European and North American potato (Simko et al., 2006; Vos et al., 2017; Sharma et al., 2018), which is comparable to our results in the diverse germplasm. The LD decay results (at a distance of ~4Mb) in our germplasm totally agree with previous studies on the global potato gene pool. LD is vital for GS breeding and its pattern may influence prediction accuracy (Jannink, 2010); however, moderate LD estimates observed in our study indicate good prediction accuracies using this population.

We scanned for associations between SNPs and traits using Q+K linear mixed model (GWASpoly) for genome-wide associations in auto-polyploids to identify genomic regions associated with late blight and PCN resistance in potatoes. We used “additive” and “X-dom” marker-effect models, which take into effect the allele dosage for tetraploid genotypes. Generally, the number of QTLs are identified for a trait in different environments and the effect of QTLs by environment interaction is erratic. In previous GWAS studies, many QTLs for late blight have been identified on different chromosomes (Juyo Rojas et al., 2019; Lindqvist-Kreuze et al., 2021). We also detected QTLs for late blight on chromosomes 11 and 8 using a complete marker data set and on chromosomes 3, 4, 5 and 11 in a reduced marker data set. The highest number of SNPs mapped on chromosome 11 indicates it as the hotspot for late blight resistance in potatoes. Earlier studies have also shown chromosomes 11 and 5 as the hotspots for resistance genes in potatoes (Pel et al., 2009). Chromosome 11 harbours major late blight resistance genes R3, R5, R6, R7, R8, R9, R10, and R11 from the *Solanum demissum*, which reside close to each other (Pel et al., 2009). Besides chromosome 11, we also observed one QTL on chromosome 5, which harbours the R1 gene for late blight resistance (El Kharbotly et al., 1994; Pel et al., 2009). Likewise, a QTL on chromosome 3 has been recently mapped for late blight resistance using multi-location data across countries (Lindqvist-Kreuze et al., 2021). Chromosome 4 harbours a R2 gene for late blight resistance in the potato and we also spotted a QTL on chromosome 4 (Destefanis et al., 2015). Besides genes from *S*. *demissum*, many other resistant genes from other species, such as S. *berthaultii* (chromosome 10), *S*. *bulbocastanum* (chromosomes 8, 6 and 4), *S*. *pinnatisectum* (chromosome 7), *S*. *mochiquense* (chromosome 9), *S*. *phureja* (chromosome 7, 9, 12) have been identified. However, *S*. *demissum* is the major species used for transferring the resistance for late blight in the cultivated species, *S*. *tuberosum*. This is evident from our study’s QTLs mapped on chromosomes 3, 4, 5 and 11, which mainly harbours the *S*. *demissum* resistant genes.

Most of the known DNA markers for *G*. *pallida* and *G*. *rostochiensis* do not show association with the phenotype data as most of these R genes/QTLs have been developed using specific segregating populations and are pedigree-specific (Gavrilenko et al., 2021). Therefore, new QTLs identified using naturally diverse global populations with varying degree of disease resistance and high-density SNPs could be potentially valuable as new markers for PCN resistance breeding. To our knowledge, no single study on QTL identification using GWAS for PCN resistance exists. In this study, we report several genomic regions associated with resistance to both species of PCN. QTLs for *G*. *pallida* resistance were found on chromosomes 1, 5 and 10 using both the marker datasets, while *G*. *rostochiensis* QTLs were located on chromosomes 4 and 10 using a complete marker dataset, and on chromosomes 1, 2, 5 and 12 using reduced marker data set. Most PCN resistant genes and QTLs have been mapped on chromosome 5, a hotspot for resistance to all major potato pathogens (Gartner et al., 2021). Most of these genes are nucleotide-binding, leucine-rich-repeat (NB-LRR) family members, which play a key role in pathogen defence within the plant kingdom. Chromosome 5 harbours different resistance genes and QTLs, namely *H2*, *Gpa^V^_spl_*, *Gpa*, *GpaM1*, *GpaV* and *Pa2/3_A* for *G*. *pallida* and *H1*, *GroVI*, *Grp1*, *Ro2_A* and *Ro2_B* for *G*. *rostochiensis* (Yang et al., 2017; Gartner et al., 2021).

Similarly, a QTL *Pa2/3_B* on chromosome 10 from *S*. *vernei* for *G*. *pallida* and *Hero* on chromosome 4 and *Gro 1.2* on chromosome 10 for *G*. *rostochiensis* resistance are previously known (Ernst et al., 2002; Park et al., 2019). However, the QTLs on chromosomes 1, 2 and 12 seem to be a new addition in the resistant QTLs/genes identification in potatoes for PCN resistance. The genetic architecture of various complex traits, such as late blight (Lindqvist-Kreuze et al., 2021; Wang et al., 2021) has also been examined previously; however, the PCN trait has been considered for the first time for GWAS.

The Q-Q plots for both the traits suggested that the false-positive marker-trait associations were effectively controlled for the genome-wide association in this study. However, the identified loci may not be the actual candidates due to LD or population structure causing false positives. Therefore, we further investigated the associations for candidate genes in the potato genome (**Table 3**). Based on functional annotation, the candidate genes for late blight were in the group of nucleotide binding and nucleic acid metabolism, response regulators (kinases), regulatory proteins in the cell signalling system, and methyltransferases, which have important implications in various disease processes (Struck et al., 2012). Besides, dentin sialophosphoprotein-related genes were also found as candidates for late blight resistance, which have been associated with metal toxicity in *Arabidopsis* earlier (Fu et al., 2014). Similarly, the plant U box proteins were associated with late blight resistance, which plays diverse roles, including immune response to stress (Trujillo, 2018). Another important superfamily is ENTH/VHS/GAT family, which belongs to membrane trafficking effectors and is required for metabolic uptake, cell growth and development (De Craene et al., 2012).

The candidate genes for *G*. *pallida* were suppressor of auxin resistance 1, SIT4 phosphatase-associated family protein, auxin response factors and intergenic modifiers, which play an important role in hormone signalling and development (Parry et al., 2006; Li et al., 2016; Máthé et al., 2021). Similarly, the *G*. *rostochiensis* candidate genes were ubiquitin-protein ligase, which directly affects biotic stress tolerance (Mazzucotelli et al., 2006) and the phosphoglycerate mutase-like family protein, which plays a critical role in stomatal movement and vegetative growth (Zhao and Assmann, 2011). The response regulators, which serve as both positive and negative regulators of the signalling mechanism and cellulose synthase-like D1, are involved in root hair development in rice (Kim et al., 2007). The important families were myb, NB-ARC domain and remorin family proteins that regulate the activities of resistant proteins (Raffaele et al., 2007; van Ooijen et al., 2008). We also found some genes with unknown functions and intergenic modifiers responsible for resistance to both the species of PCN.

The genomic prediction estimates showed high accuracy for late blight resistance (0.42-0.51), while resistance to both the species of PCN (0.04-0.21) observed very low prediction accuracy. The results were encouraging to implement genomic selection for late blight in potato breeding programs, but the PCN trait requires further improvement in prediction accuracy by varying one or the other factors affecting genomic selection. Similar prediction accuracy (0.41-0.86) for the late blight trait has been reported in earlier studies in potatoes (Enciso-Rodriguez et al., 2018; Stich and Van Inghelandt, 2018; Lindqvist-Kreuze et al., 2021). The PCN trait, however, was considered for the first time to estimate genomic prediction accuracy in potatoes in this study.

The genomic prediction accuracy results were similar for the complete vs reduced number of markers, explaining that the number of markers might not affect the prediction accuracy (Sverrisdóttir et al., 2018, FPS). However, the markers coverage should be sufficient enough based on LD decay and QTLs must be in LD with at least one marker, therefore capturing the majority of the genetic variance (Slater et al., 2016). In our case, the reduced marker data set had 24,000 markers spread across the potato genome, which was much higher than the minimum number of 8,000 to 15,000 SNPs calculated earlier (Slater et al., 2016; Sharma et al., 2018)

The second most important factor is trait heritability, which influences the prediction accuracy for most of the traits. However, there was no correlation between trait heritability and prediction accuracy for *G*. *pallida* and *G*. *rostochiensis* resistance in our study. Moderate to high heritability also resulted in low prediction accuracy for PCN resistance, while similar heritability estimates for late blight resistance showed high prediction accuracy. This could be attributed to the size of the training population, as to get meaningful prediction accuracies for traits with low heritability, a considerably larger training population is necessary (Slater et al., 2016). An increase in training population size shows a corresponding increase in genomic prediction accuracy for complex traits in potatoes (Slater et al., 2016), but smaller training populations of comparable size have also revealed moderate to high prediction accuracies (Caruana et al., 2019; Sood et al., 2020b; Sood et al., 2020c). Nevertheless, a subset of populations with very small size reported low prediction accuracies (Sverrisdóttir et al., 2018).

## Conclusion

Genotypic and phenotypic evaluation of 222 diverse potato accessions identified QTLs for late blight and PCN traits. For late blight, 8 QTLs, while 9 and 13 for *G*. *pallida* and *G*. *rostochiensis* were found in our study. The predominant QTLs were on chromosome 11 for late blight, while chromosomes 1, 5 and 10 were the hotspots for PCN resistance. The genomic prediction accuracy for late blight was high, whereas PCN observed low prediction accuracy. The low genomic prediction accuracy for the PCN trait could be attributed to its highly complex genetic inheritance, which might require a large reference population and robust phenotypic observations. The results revealed that the identified genomic regions and candidate genes need functional validation. The MTAs and genomic prediction results could be integrated into the biotic stress resistance potato breeding program for enhanced genetic gain in developing new resistant cultivars.

## Data availability statement

The datasets presented in this study can be found in online repositories. The names of the repository/repositories and accession number(s) can be found below: https://www.ncbi.nlm.nih.gov/, PRJNA723185.

## Author contributions

Conceptualization: SSo. Investigation – genotyping: SSo, AK and VB. Investigation - glass house/field experiments: SSo, VB, SSh, AB, D, AS, ML. Data curation and analysis: SSo. Writing - original draft: SSo. Editing: SSo and VK. All authors contributed to the article and approved the submitted version.

## Supplementary material

Supplementary Figure 1 The experimental procedure of DNA library preparation for Genotyping by Sequencing.

Supplementary Figure 2 Relationship between AUDPC values of different accessions across the years **(A)** 2018 and 2019 **(B)** 2019 and 2020 **(C)** 2018 and 2020.

Supplementary Figure 3 Relationship between PCN grades of different accessions across the years **(A)** *Globodera pallida* scores relationship in the year 2020 and 2021 **(B)** *Globodera rostochiensis* scores relationship in the year 2020 and 2021. GP2020- Grading of accessions for resistance to *G. pallida* in the year 2020; GP2021- Grading of accessions for resistance to *G*. *pallida* in the year 2021; GR2020- Grading of accessions for resistance to *G. rostochiensis* in the year 2020; GR2021- Grading of accessions for resistance to *G. rostochiensis* in the year 2021.

Supplementary Figure 4 Chromosome-wise SNP number identified through GBS in the potato genome.

Supplementary Figure 5 Chromosome-wise LD in all the 12 chromosomes of potato. The chromosome number is indicated on the top of the each sub-figure. The green lines shows the SNPs position in the genome. D`colour key shows the linkage disequilibrium with in the chromosome. The areas with red colour reflect high linkage disequilibrium regions of the chromosome.
