# Supplementary figures and images for: Genome-wide association mapping and genomic prediction for late blight and potato cyst nematode resistance in potato (Solanum tuberosum L.)
Source: Front Plant Sci. 2023 Oct 4;14:1211472. doi: 10.3389/fpls.2023.1211472 (PMC10582711; doi:10.3389/fpls.2023.1211472)

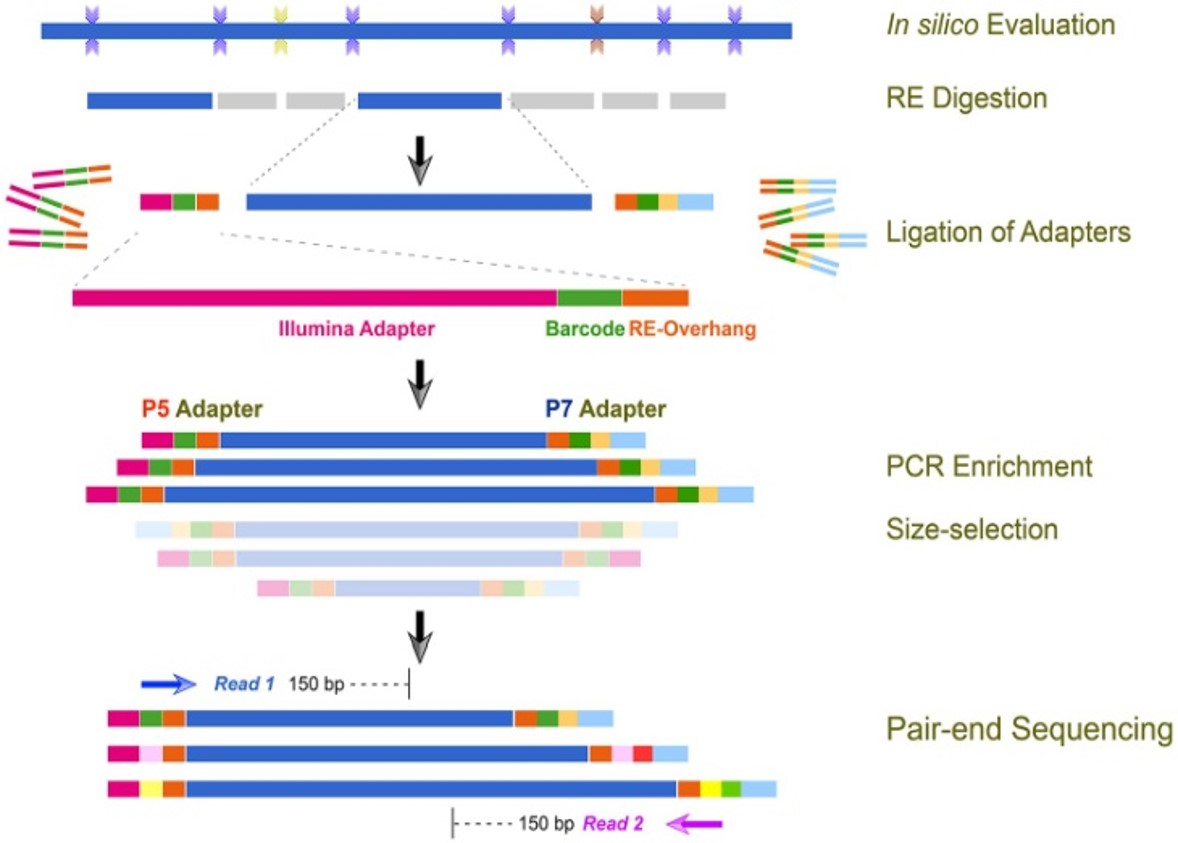

Supplement: Supplementary Figure 1 — The experimental procedure of DNA library preparation for Genotyping by Sequencing. [file Image_1.jpeg]

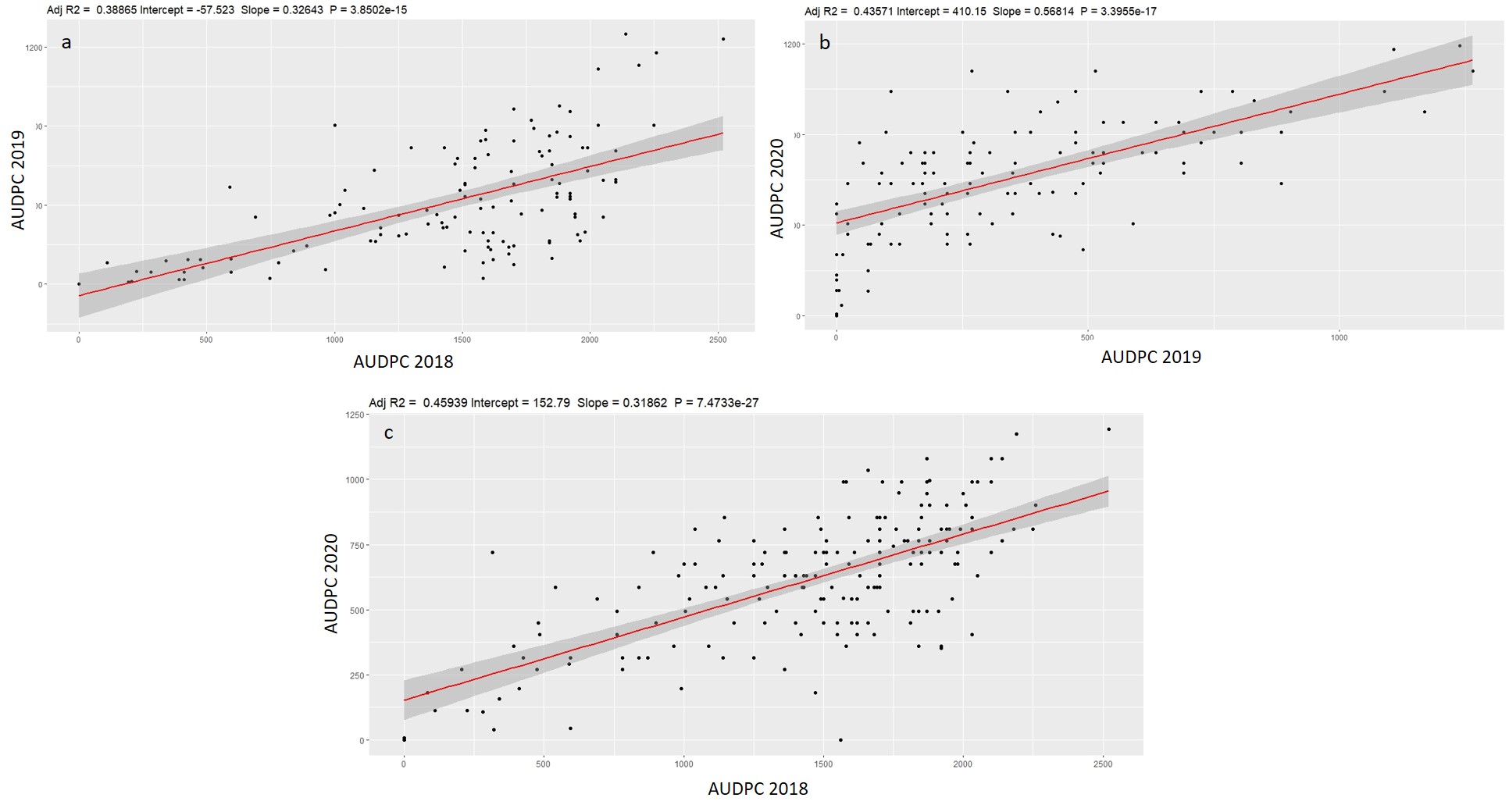

Supplement: Supplementary Figure 2 — Relationship between AUDPC values of different accessions across the years (A) 2018 and 2019 (B) 2019 and 2020 (C) 2018 and 2020. [file Image_2.jpeg]

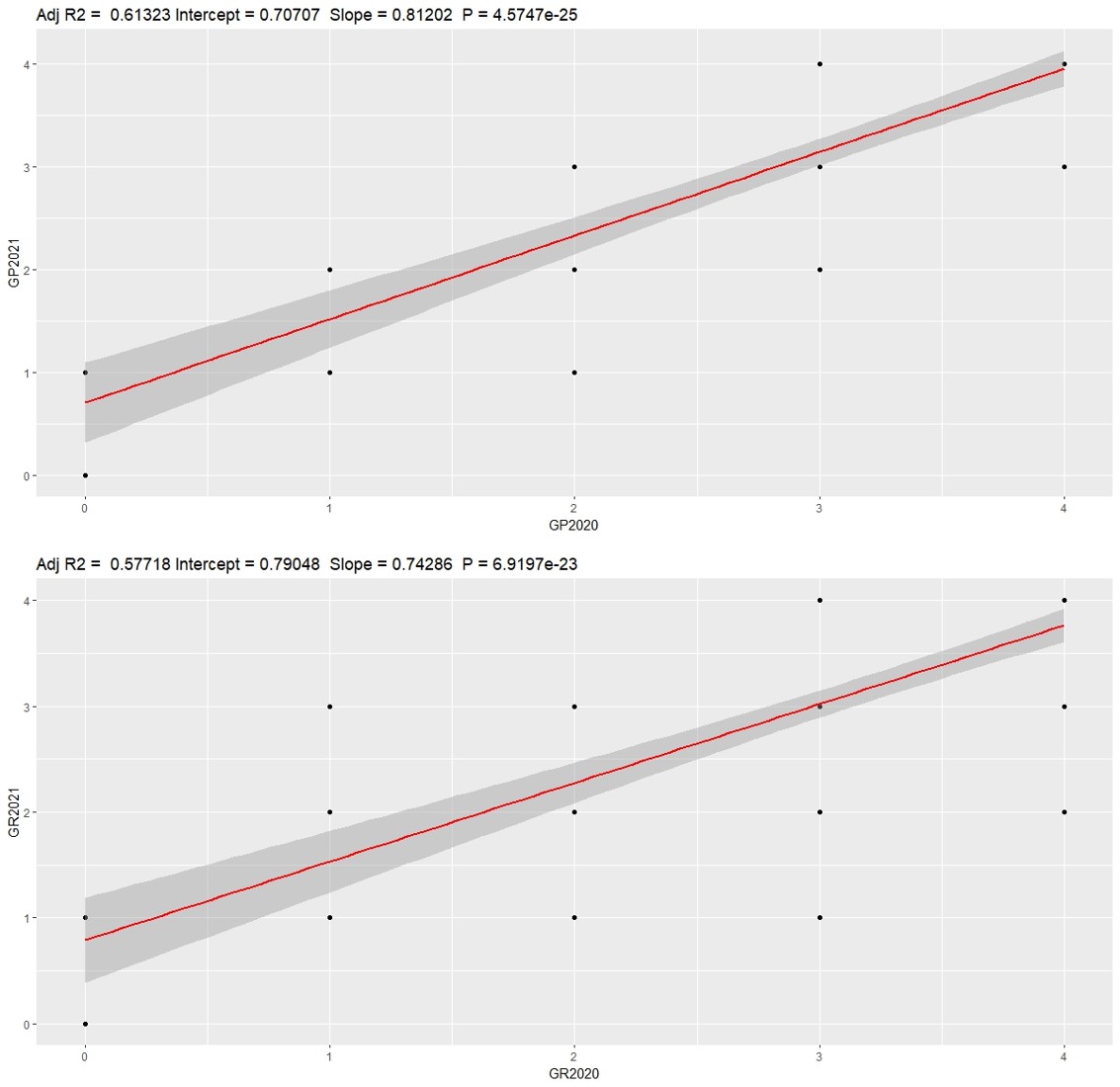

Supplement: Supplementary Figure 3 — Relationship between PCN grades of different accessions across the years (A) Globodera pallida scores relationship in the year 2020 and 2021 (B) Globodera rostochiensis scores relationship in the year 2020 and 2021. GP2020- Grading of accessions for resistance to G. pallida in the year 2020; GP2021- Grading of accessions for resistance to G. pallida in the year 2021; GR2020- Grading of accessions for resistance to G. rostochiensis in the year 2020; GR2021- Grading of accessions for resistance to G. rostochiensis in the year 2021. [file Image_3.jpeg]

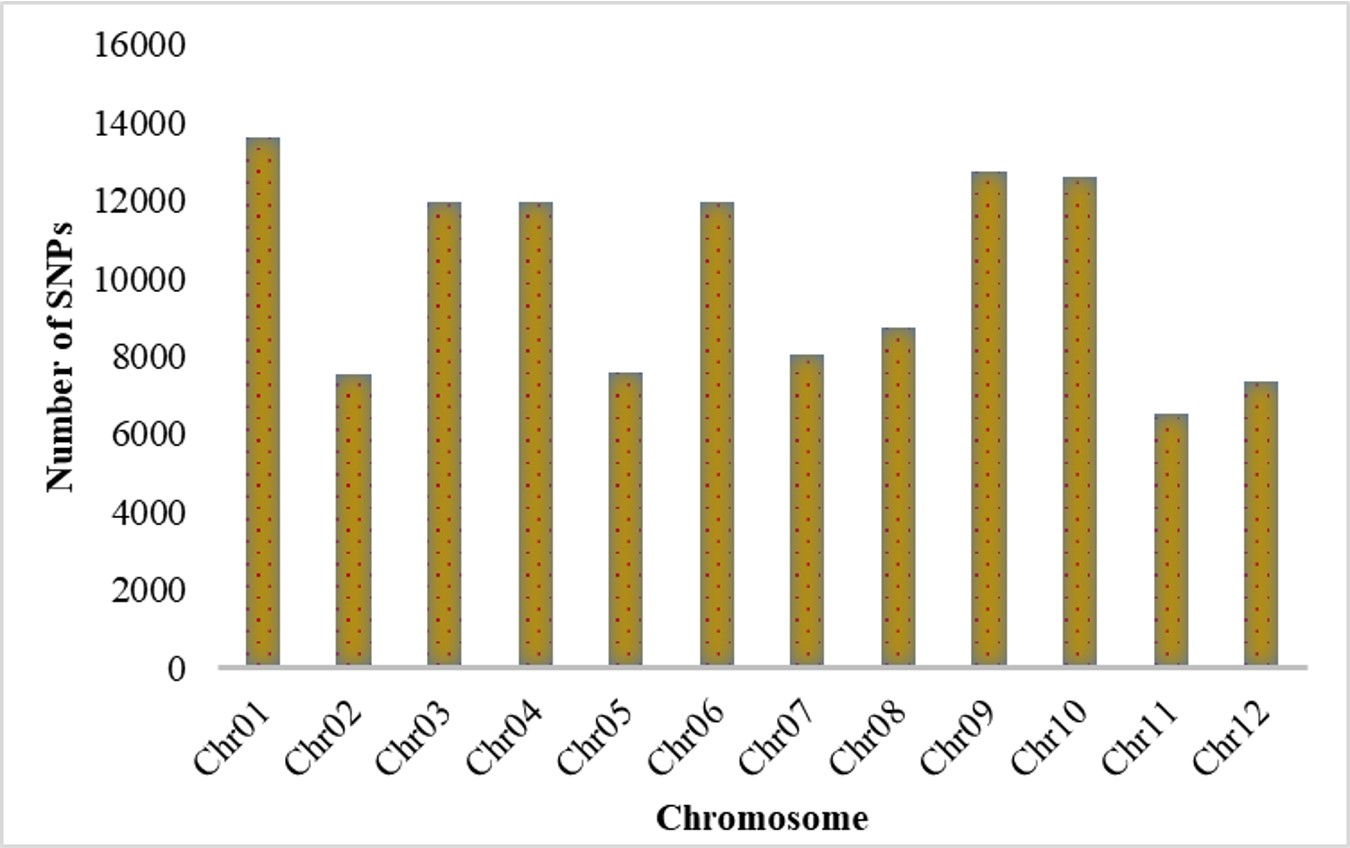

Supplement: Supplementary Figure 4 — Chromosome-wise SNP number identified through GBS in the potato genome. [file Image_4.jpeg]

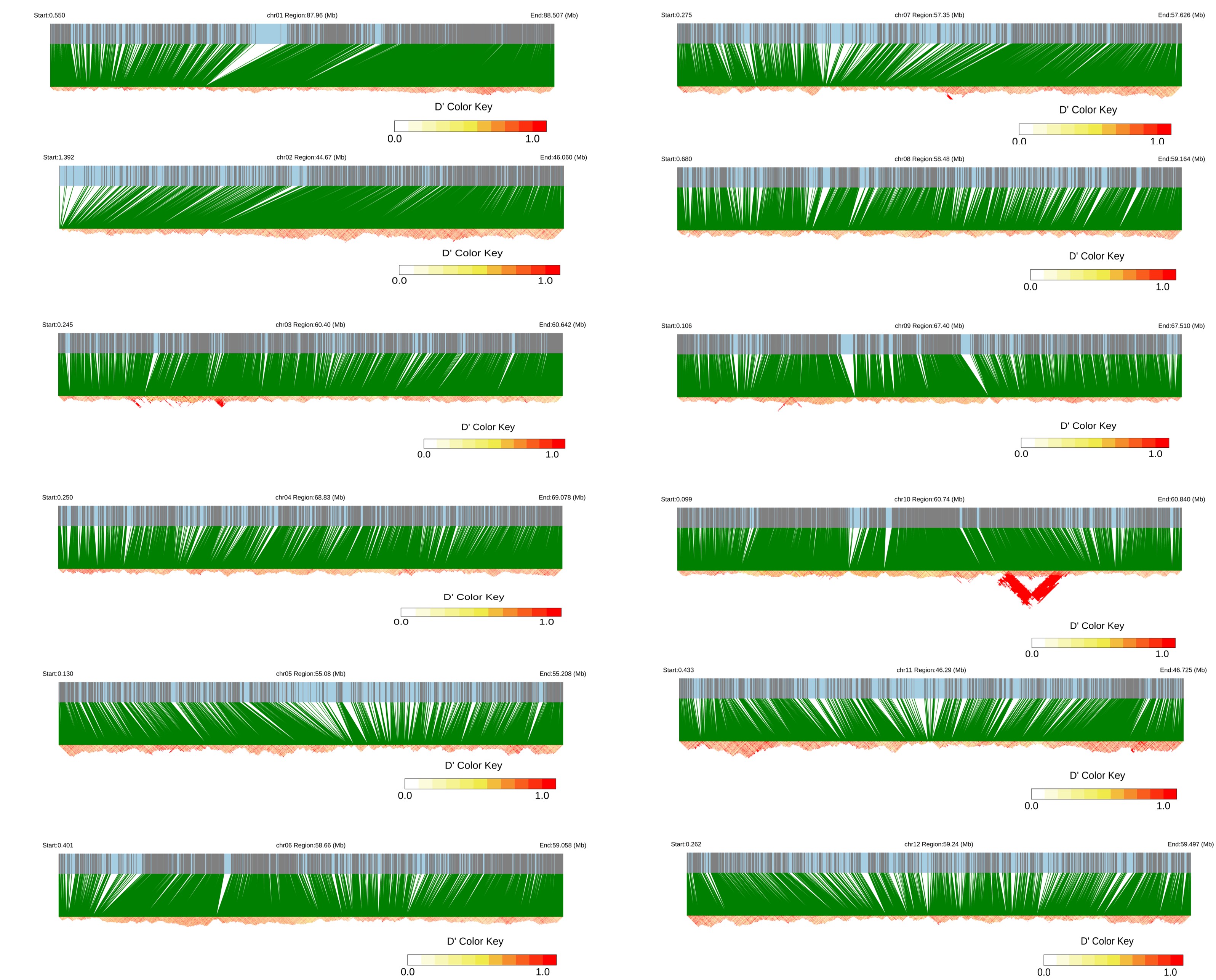

Supplement: Supplementary Figure 5 — Chromosome-wise LD in all the 12 chromosomes of potato. The chromosome number is indicated on the top of the each sub-figure. The green lines shows the SNPs position in the genome. D`colour key shows the linkage disequilibrium with in the chromosome. The areas with red colour reflect high linkage disequilibrium regions of the chromosome. [file Image_5.jpeg]
